# Nutrient Intakes from Meals and Snacks Differ with Age in Middle-Aged and Older Americans

**DOI:** 10.3390/nu11061301

**Published:** 2019-06-08

**Authors:** Jessica L. Krok-Schoen, Satya S. Jonnalagadda, Menghua Luo, Owen J. Kelly, Christopher A. Taylor

**Affiliations:** 1 Division of Health Sciences, School of Health and Rehabilitation Sciences, College of Medicine; Comprehensive Cancer Center, The Ohio State University, Columbus, OH 43210, USA; Jessica.Krok@osumc.edu; 2 Abbott Nutrition, Nutrition Science, Columbus, OH 43219, USA; satya.jonnalagadda@abbott.com (S.S.J.); menghua.luo@abbott.com (M.L.); owen.kelly@abbott.com (O.J.K.); 3 Division of Medical Dietetics, School of Health and Rehabilitation Sciences, College of Medicine, The Ohio State University, Columbus, OH 43210, USA; 4 Department of Family Medicine, College of Medicine, The Ohio State University, Columbus, OH 43210, USA

**Keywords:** breakfast, lunch, dinner, aging, NHANES, dietary intakes, dietary patterns, middle-aged adults, meal patterns, snack intakes

## Abstract

The present study investigated the meal patterns across demographic characteristics in middle-aged and older US adults. Study participants were noninstitutionalized participants from the 2005–2016 National Health and Nutrition Examination Survey, an observational cross-sectional study. Data from 17,361 adults were categorized into 45–59 years (*n* = 7366), 60–70 years (*n* = 5348), and 71+ years (*n* = 4647) to compare demographics, nutrient intakes, and meal patterns. Dietary recalls were collected using the multiple-pass method. Data analyses were weighted to create a nationally representative sample. Two-thirds of adults reported consuming three meals on the day of intake. Lunch was the most often skipped meal across all age groups. A greater proportion of adults over 70 years reported consuming breakfast, while a smaller proportion reported consuming snacks. Significant differences were observed in total energy and nutrient intakes and proportion of the day’s intakes by meal. Grain, milk, and dairy food group intakes were highest at breakfast, while the protein food group intakes were highest at lunch and dinner. Age-related differences in meal consumption and composition provide valuable formative data to support targeted nutritional education and intervention opportunities to promote and encourage healthy food choices.

## 1. Introduction

Diet and nutrition play important roles in supporting healthy aging. Generally, dietary guidelines for aging individuals are focused on meeting nutrients of concern, and modifying dietary intake patterns to address their changing health and nutritional status, such as alterations in metabolism, digestion and absorption, and lifestyles. Sufficient nutrient intakes are also critically important for middle-aged and older adults as aging is associated with increased risk of chronic diseases, such as cardiovascular disease and diabetes, and sarcopenia, a gradual and progressive decline in muscle mass, strength, and endurance [1,2]. Mounting evidence shows that the increasing prevalence of these conditions at younger ages is not a normal function of aging, but rather a consequence of unhealthy behaviors [3,4,5]. Therefore, meeting the nutrient needs through healthy dietary patterns is key to maintaining good health for healthy aging.

Existing evidence suggests that older adults are consuming less food, which contributes to suboptimal amounts of energy and key nutrient intakes [6] and this is associated with development of chronic conditions. The etiology of suboptimal dietary intakes is complex and related to several factors such as predicted reduced energy needs, poor appetite (age- and disease-related anorexia), dysphagia, dental issues, physical and mental disabilities that limit acquiring and preparing food, changes in food preferences and food choices, changes in meal patterns and meal skipping, and food insecurity due to financial and social limitations [7,8].

Dietary intake patterns among all Americans have shifted unfavorably toward higher total energy intakes, more frequent snacking, and higher intakes of sweetened beverages, especially among younger adults [9,10,11,12], contributing to shifts in food group and nutrient intakes. The behavior shift may be continued to older age by those in the younger age groups. Between 1977 and 2006, among adults aged >60 years, snacking occasions and total energy intakes increased, while shifts towards low-fat meat and dairy food intakes were observed [12]. While examining protein intakes, the oldest adults who met protein recommendations (0.8 g per kg of body weight per day) on the day of intake had an overall better diet quality than middle-aged adults, yet intake fell short of the recommendations of the Dietary Guidelines for Americans [13].

With an increase in snacking intakes, limited information is available on current meal intake patterns and their contributions to total dietary intakes among middle-aged and older American adults [1,14]. Meal skipping and snack intake patterns can influence nutrient intakes, risk of chronic disease, and aging-associated declines in functional outcomes [5,15]. This is increasingly relevant as quality of life among older adults is an important consideration among this growing segment of the American population. Understanding age-related differences in meal intakes could be pivotal for targeted nutritional interventions to promote optimal health with aging. Therefore, the purpose of this study was to examine meal and snack intake patterns and their contributions to macronutrient intakes among a nationally representative sample of middle-aged and older adults.

## 2. Materials and Methods

Dietary data from 17,361 adults aged 45 years and older from the 2005–2016 National Health and Nutrition Examination Survey (NHANES) were examined to assess the differences in age-related meal and snacking intakes. NHANES is a national nutrition surveillance system that assesses the health and nutritional status of the noninstitutionalized population in the US. Specific populations were oversampled to improve representation in the data, including those from low-income households, racial and ethnic minorities, and older adults. Demographic and personal characteristic data were collected during the in-home interview and the dietary intake data were collected during the mobile examination center visit. All data collection procedures were approved by the Research Ethics Review Board of the Center for Disease Control and Prevention’s National Center for Health Statistics.

### 2.1. Dietary Intake Data

Assessment of dietary intakes was conducted using the first day of dietary recall data collected in the mobile examination center using the automated multiple-pass method [16]. Trained interviewers used the computer-assisted dietary interview system to obtain all foods and beverages consumed during the previous day, between midnight and midnight. Energy and nutrient intakes were estimated using the Food and Nutrition Database for Dietary Surveys [17] and the Food Patterns Equivalents Database [7]. Nutrient intakes were obtained for the day of intake to represent total intakes. To account for differences in total energy intakes, energy-adjusted nutrient intakes were computed to present intakes per 1000 kcal.

To assess meal intakes, participants were asked during the interview to provide the time of day and self-disclosed the names of the eating occasions. Eating occasions were classified as breakfast, lunch, dinner, and snacks based on the What We Eat in America meal coding strategy [18]. Meal consumption was determined if the participants reported any food or beverages for the corresponding meal or snacking occasion. The total number of meals was obtained as the presence or absence of breakfast, lunch, and dinner; snacking occasions were not included in the total number of meals. Macro and micronutrient intakes from meals and snacks were estimated by aggregating the nutrient intakes across all food consumed for the corresponding meal or snack. The proportion of the day’s intake obtained at eating occasions was computed as the energy and nutrient intakes from the specified eating occasion divided by the total energy and nutrient intakes for all foods and beverages reported on the day of record.

To evaluate the types of foods that contributed to total energy intake during meals and snacking occasions, energy intakes were aggregated by the What We Eat in America food categories from the individual foods reported during the dietary recall. The proportion of the eating occasions energy obtained from each food category was divided by the total amount of energy obtained from that meal or snack within each age category. Using breakfast as an example, the percent of energy from breakfast obtained from milk and dairy products was computed as the proportion of all of the energy from milk and dairy products divided by all of the energy consumed at breakfast.

### 2.2. Statistical Analysis

To assess differences in dietary intakes across age groups, participants were stratified into three age categories for analysis: 45–59 years, 60–70 years, and ≥71 years. Public use data files were downloaded from the National Center for Health Statistics (NCHS) website and imported into SPSS Complex Samples (version 25, IBM, Armonk, NY, USA 2018) for tabulation. Pearson’s Chi-Square was used to assess significant differences in the reporting of meals and snacks by age categories. Analysis of covariance, controlled for gender, race/ethnicity, household income, percent of the federal poverty rate, and marital status, were conducted to assess mean differences in total and energy-adjusted nutrient intakes by eating occasion. Descriptive data were presented as means and standard errors, as well as unweighted sample size and the weighted population percent. The sample was weighted using the dietary sample weight provided by NCHS in accordance with the analytic guidelines to produce nationally-representative estimates of food and nutrient intakes. This procedure accounts for the complex sampling design used to collect the sample to produce a nationally representative sample while producing appropriate standard errors that reduce type 1 error in statistical testing.

## 3. Results

Complete dietary data on the day of intake for adults aged 45 years and older were categorized into 45–59 years (*n* = 7366), 60–70 years (*n* = 5348), and ≥71 years (*n* = 4647). Approximately half of the sample was female, and two-thirds were married or living as married, but among adults over 70 years of age, fewer were married (54.8%) and more were female (57.8%). The proportion of those who were non-Hispanic white was highest in the oldest age group, 71 years or older (82%), compared to those 60–70 years (76.5%) and 45–69 years (70.7%); data not shown.

### 3.1. Meal and Snack Intakes

In all age groups, lunch was the most likely skipped meal on the day of intake. There was no significant difference in the proportion of adults consuming lunch by age category (Table 1). Adults over 70 years had a significantly greater proportion reporting breakfast consumption and significantly smaller proportion reporting snacking occasions (*p* < 0.001, respectively), compared to the other age groups. Approximately two-thirds of adults, regardless of age, reported consuming three meals on the day of intake.

### 3.2. Mean and Percent Macronutrient Intakes from Meals and Snacks

Significant differences in total energy and nutrient intakes and proportion (%) of the day’s intakes by meal are presented in Table 2 and Table 3 respectively. Adults 71 years or older had the lowest total daily energy (1751 kcal) and added sugar intakes (47.4 g). Adults 71 years or older consumed significantly more energy from breakfast compared to the 45–59 year old age group (398 vs. 373 kcal; *p* = 0.009), accounting for 23.4% of the day’s energy intake, which was significantly different from all other age groups (*p* < 0.001). For lunch, dinner, and all snacks, energy intakes significantly decreased as age increased; however, in relation to percent daily energy intake only all snacks were significantly different between all groups.

Protein intakes (g) at breakfast did not significantly differ between age groups; however, percent protein intake at breakfast significantly increased as age increased (*p* < 0.001). Protein intakes (g) for other meals and all snacks were significantly lower as age increased (*p* < 0.001). However, for percent protein intake, dinner and all snacks were significantly lower in the youngest versus oldest age group (*p* = 0.033) and protein intake for all snacks was also significantly lower in the 71+ year old group compared to the 60–70 year old group (*p* < 0.001). More than one-third of the day’s total fat intakes were consumed during dinner, with significant differences in the mean quantity of fat consumed, but not the proportion (%) of the day’s fat intakes. Adults 71 years or older had significantly lower mean intakes of total fat and saturated fat from snacks, as well as a lower proportion of the day’s fat obtained from snacks (*p* ≤ 0.001).

Older adults consumed significantly less carbohydrates from snacks than their younger counterparts (*p* < 0.001) on the day of intake. Older adults had a significantly greater contribution of carbohydrate and added sugar intakes from breakfast (*p* < 0.001), but a significantly smaller proportion of the day’s energy from snacks (*p* < 0.001). As well, added sugars intakes were significantly lower in the older adults (*p* < 0.001), while during meals (breakfast, lunch, and dinner), mean added sugars intakes were significantly higher for the 45–59 year group compared to the 60–70 and 71+ age groups (*p* ≤ 0.001). Fiber showed mixed results, but breakfast intakes were significantly higher in the oldest adults for both mean and percent intakes (*p* < 0.001).

With significant differences in the energy and nutrient intake estimates across meals by age, energy-adjusted intakes were compared to assess nutrient density per 1000 kcal (Table 4) by age. Adults 71 years or older had a significantly lower density of protein but a significantly higher density of carbohydrate at breakfast and dinner. Added sugars content was the highest in breakfast, lunch, and snacks in the 45–59-year-olds (*p* < 0.001), while mean fiber density was significantly poorer in all meals and snacks (*p* < 0.001).

### 3.3. Food Sources of Energy from Meals and Snacks

**Figure 1:**
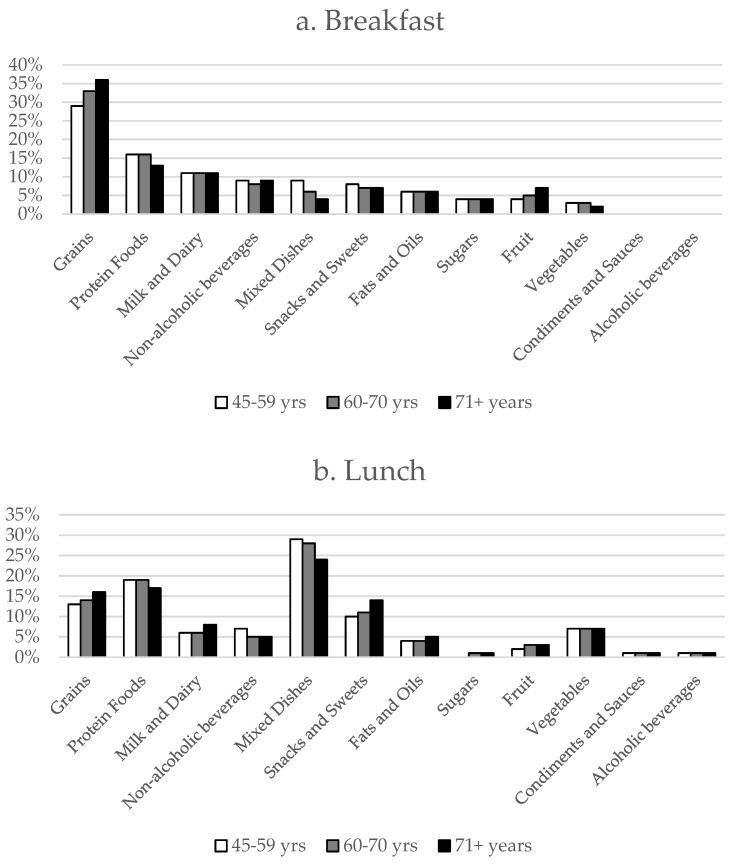

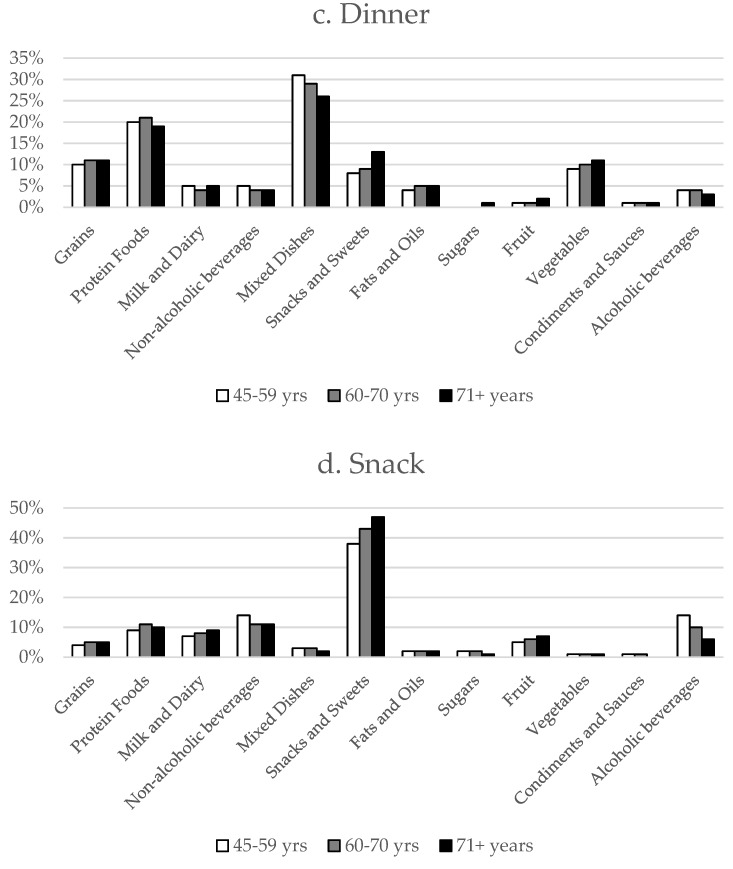
Percent of energy from food sources based on the US Department of Agriculture What We Eat in America food categories during meals and snack occasions by age categories during (**a**) breakfast, (**b**) lunch, (**c**) dinner, and (**d**) snacks.

To understand what foods are contributing to meal intakes across the age groups, the percent of energy obtained from food groups during meals and snacks were estimated (Figure 1a–d). Grains, protein foods, and milk and dairy foods accounted for over half of the energy consumed at breakfast, with the greatest proportion of energy from grains in adults over 70 years. Mixed dishes contributed the most to lunch and dinner intakes, with greater contributions in the younger age groups, followed by protein foods. Snacks and sweets accounted for approximately one-third to one-half of energy intakes from snacks, with the greatest contribution shown in adults over 70 years.

## 4. Discussion

Evaluation of the meal and snack intake patterns of Americans of middle-aged and older adults and their contribution to overall dietary intakes on the day of intakes offer valuable formative data to support nutrition education efforts and more intervention targets to promote healthful aging. Eating occasions afford new opportunities to meet the day’s needs and numerous factors influence the intakes in older adults [19,20]. Data from NHANES III (1988–1994) estimated less than two-thirds of adults over 65 years ate three meals on the day of record, with greater frequency of eating related to higher intakes of carbohydrates, fiber, and some micronutrients, as well as lower intakes of protein, fat, and sodium [21]. Middle-aged adults with three or fewer eating occasions per day had significantly higher percent of energy from protein and lower percent of energy from carbohydrates than those with more eating occasions [22]. There is support for the frequency of consumption, but more is needed on the potential health impact of missed meals, especially in aging. Including the foods typically consumed during eating occasions will help provide valuable formative data to inform strategies to optimize dietary intakes in older adults.

There is a paucity of data exploring the meal patterns of middle-aged and older adults, and challenges persist with the classification of intakes from a methodological standpoint [14]. Leech et al. [14] observed lunch and evening meals to contribute to the greatest proportion of total daily energy, protein, fat, and carbohydrate intake of adults from the UK, and main meals were where the largest volume of food was normally consumed. As well, those with a greater proportion of intakes later in the day were related to poorer diet quality and greater body mass index (BMI) [23]. Data from the present study indicate that dinner was the least skipped meal on the day of intake and was the main contributor to energy and most macronutrients for all ages in the U.S. population. In contrast, Howarth and colleagues [24] found that breakfast was the least skipped meal of the day among older adults and that older adults skipped fewer meals compared to younger adults. In the current study, lunch was the most commonly missed meal, with lower intakes of energy, protein, and fat in older adults, and lower in carbohydrate compared to the 45–59 year old group. Howarth et al. [24] also found significantly lower intakes of energy and fat from lunch among older adults, yet a higher intake of protein compared to younger adults. In an analysis by Aljuraiban et al., adults who ate more frequently throughout the day presented with a greater proportion of intakes earlier in the day and had lower energy density and total energy [23].

There is a great emphasis on breakfast consumption, noting that older adults are more likely to consume breakfast [24,25,26]. Interventions to address breakfast and lunch intakes in older adults have produced significant improvements in nutritional intakes and quality of life measures [27,28]. From the present study, older adults presented a greater percent contribution of the day’s total energy, macronutrient, fiber, and added sugars from breakfast than their younger counterparts, corresponding with Howarth and colleagues [24] findings. Older adults who consumed more whole grains, low-fat dairy, and fruit, commonly associated with traditional breakfast intakes, had less insulin resistance and inflammation [29]. As the traditional American breakfast contributes to grains, fruit, total fiber, folate, and calcium intakes, especially in older adults, the implications of breakfast skipping in older adults needs further exploration.

With a primary focus on meal intakes, the contribution of snacking occasions to overall intakes in older adults is meager and conflicting [30]. Despite the evidence that snacks are commonly perceived as less healthy options [31] as also seen in the present study, in adults 20 years and older, higher snacking frequency was linked to significantly better diet quality scores [32,33,34]. In contrast, Leech et al. [14] showed that snacking among US adults >65 years contributed to higher protein, carbohydrate, and fat intake. The present study agrees with previous research [24,34], showing older adults had the lowest intakes from snacks compared to their younger counterparts.

Nutritionally, snacking intakes have been shown to account for approximately 10–20% of micronutrient intakes in adults over 65 years [33]. For middle-aged and older adults in the present study, snacking occasions contributed 17–22% of estimated energy intakes on the day of intake, and were comprised of more energy-dense, nutrient-poor food choices. Snacks contributed the greatest proportion of added sugar intakes than did the three main meals, with nonalcoholic beverages and snacks and sweets foods accounting for nearly half of the energy from snacks. The presence of poorer snacking food choices and the greater contribution to total intakes in 45–59 year old adults from the present study warrant further examination.

These data suggest there is an opportunity to help improve the nutrient intakes at meal and snack times in middle-aged and older adults, with strategic opportunities to improve overall diet quality. Finding ways to enable older individuals to increase intake of more nutrient-dense meals and snacks and limit meal skipping can help them meet their daily dietary and nutrient intake goals [35]. Age-associated decline in appetite is a common observation, hence smaller meals and snacks that have enhanced sensory and taste attributes, access to more variety of nutrient-dense foods at meal and snack times, and supplemental nutrient enrichment of foods can help improve dietary and nutrition intake. Further, the distinctive differences in eating patterns noted in the middle-aged adults (45–59 years) suggest that dietary alterations to promote successful aging are warranted earlier in the lifecycle.

### Strengths and Limitations

The present study has several strengths and limitations that need to be considered when assessing the results. This study utilizes data from a large national nutrition monitoring surveillance program, producing a nationally representative estimate of US adult’s dietary intakes. Differences in proportional intakes by meal and snack intake patterns were assessed, which identifies opportunities for nutrition intervention targets. While national surveillance data are useful to assess broader dietary patterns, study limitations also need to be acknowledged. This cross-sectional population intake analysis is based on a single 24-hour dietary recall; although the multiple pass method helps to maximize recall and accuracy of the data collected [36], it is still reliant on self-reported dietary intake. These reported intakes are associated with the known limitations of dietary recalls [37], including underreporting across different individual demographic characteristics and food groups. Further, these data cannot be assumed to represent usual intakes. As a cross-sectional study, there is not a causal inference from the data. Future studies should examine the changes in individual meal and snack intake patterns over time and relationships to healthy aging and health outcomes, as choices at each meal ultimately determine the overall daily intake.

## 5. Conclusions

In summary, these data demonstrate that meal skipping was common on the day of intake in middle-aged and older adults, with lunch being the most skipped meal, followed by breakfast. Older adults had a greater density of carbohydrates during meals, with a lower density of protein and added sugars from meals and snacks. Helping aging individuals make the right food choices at each eating occasion will help improve nutrient intakes and potentially lead to positive health outcomes. Learning more about food choices at each eating occasion may better assist individualizing dietary advice compared to the traditional focus on total daily intakes.

## Figures and Tables

**Table 1:** Personal characteristics and proportion of adults consuming meals and snacks, categorized by age.

		Age Categories (Years)						
		45–59 (*n* = 7366)		60–70 (*n* = 5348)		≥71 (*n* = 4647)		
**Meal Data ^1,2^**	**Category**	***n***	**%**	***n***	**%**	***n***	**%**	***p***
Meal Consumed	Breakfast	6369	87.7%	4805	91.3%	4382	95.4%	<0.001
	Lunch	5533	79.8%	3849	78.2%	3372	77.1%	0.064
	Dinner	6660	93.3%	4772	93.9%	4209	93.1%	0.469
	All Snacks	6983	96.4%	5013	96.3%	4301	93.3%	<0.001
Number of Meal Occasions Reported	0	25	0.2%	22	0.3%	8	0.1%	0.007
	1	469	4.9%	328	4.1%	179	2.2%	
	2	2523	28.7%	1896	27.7%	1596	29.6%	
	3	4349	66.2%	3102	68.0%	2864	68.1%	
**Personal Characteristics ^2^**	**Category**	***n***	**%**	***n***	**%**	***n***	**%**	***p***
Gender	Male	3587	48.3%	2653	47.2%	2305	42.7%	<0.001
	Female	3779	51.7%	2695	52.8%	2342	57.3%	
Marital Status	Single/Widowed/Divorced	2600	31.8%	2025	30.7%	2215	45.2%	<0.001
	Married/Living as Married	4761	68.2%	3320	69.3%	2431	54.8%	
Race/Ethnicity	Mexican American	1125	6.4%	906	5.1%	367	3.3%	0.809
	Other Hispanic	733	4.6%	640	3.5%	275	2.5%	
	Non-Hispanic White	3112	70.7%	2090	76.5%	3047	82.0%	
	Non-Hispanic Black	1695	11.5%	1349	9.5%	717	7.7%	
	Other or Multiracial	701	6.7%	363	5.5%	241	4.6%	

^1^ Number of meals computed based on self-reported consumption of foods or beverages for breakfast, lunch, and dinner. ^2^ Significance determined using Pearson’s Chi-Square.

**Table 2:** Differences in mean intakes of macronutrients obtained at meals and snacks by age categories.

Intake	Meal	Age Categories (Years)			*p*
		45–59	60–70	≥71	
Energy (kcal)	Breakfast	373 (6)	384 (5)	398 (6)	0.009
	Lunch	502 (8)	434 (9)	407 (8)	<0.001
	Dinner	773 (10)	683 (11)	631 (11)	<0.001
	All Snacks	513 (11)	429 (11)	315 (9)	<0.001
Protein (g)	Breakfast	13.6 (0.3)	14.1 (0.2)	14.0 (0.3)	0.208
	Lunch	23.0 (0.4)	20.0 (0.4)	18.5 (0.4)	<0.001
	Dinner	37.0 (0.5)	32.5 (0.5)	29.1 (0.5)	<0.001
	All Snacks	10.8 (0.2)	9.6 (0.3)	7.2 (0.2)	<0.001
Carbohydrate (g)	Breakfast	49.6 (0.8)	51.8 (0.8)	57.9 (0.9)	<0.001
	Lunch	56.0 (0.9)	48.0 (1.1)	46.5 (0.9)	<0.001
	Dinner	79.9 (1.2)	72.2 (1.4)	69.2 (1.3)	<0.001
	All Snacks	69.1 (1.5)	57.6 (1.3)	42.6 (1.2)	<0.001
Added sugars (g)	Breakfast	12.4 (0.3)	10.9 (0.3)	10.7 (0.3)	<0.001
	Lunch	12.9 (0.4)	9.7 (0.4)	9.1 (0.5)	<0.001
	Dinner	16.4 (0.6)	13.7 (0.5)	13.7 (0.6)	0.001
	All Snacks	28.3 (0.9)	21.3 (0.7)	13.9 (0.6)	<0.001
Dietary fiber (g)	Breakfast	3.2 (0.1)	3.7 (0.1)	4.4 (0.1)	<0.001
	Lunch	4.3 (0.1)	3.9 (0.1)	3.8 (0.1)	<0.001
	Dinner	6.3 (0.1)	6.3 (0.1)	5.9 (0.1)	0.021
	All Snacks	3.4 (0.1)	3.2 (0.1)	2.6 (0.1)	<0.001
Total fat (g)	Breakfast	14.0 (0.3)	14.1 (0.3)	13.3 (0.3)	0.079
	Lunch	20.8 (0.4)	18.1 (0.4)	16.5 (0.5)	<0.001
	Dinner	31.9 (0.5)	27.4 (0.5)	25.2 (0.5)	<0.001
	All Snacks	16.8 (0.4)	15.4 (0.5)	12.0 (0.4)	<0.001
Saturated fat (g)	Breakfast	4.7 (0.1)	4.7 (0.1)	4.5 (0.1)	0.242
	Lunch	6.3 (0.1)	5.4 (0.1)	5.1 (0.2)	<0.001
	Dinner	10.2 (0.2)	8.6 (0.2)	7.8 (0.2)	<0.001
	All Snacks	5.7 (0.1)	5.1 (0.2)	4.0 (0.2)	<0.001
Monounsaturated fat (g)	Breakfast	5.1 (0.1)	5.2 (0.1)	4.7 (0.1)	0.013
	Lunch	7.5 (0.2)	6.5 (0.2)	5.9 (0.2)	<0.001
	Dinner	11.3 (0.2)	9.8 (0.2)	9.0 (0.2)	<0.001
	All Snacks	6.1 (0.2)	5.7 (0.2)	4.5 (0.2)	<0.001
Polyunsaturated fat (g)	Breakfast	3.0 (0.1)	3.1 (0.1)	2.9 (0.1)	0.577
	Lunch	5.0 (0.1)	4.6 (0.2)	4.1 (0.1)	<0.001
	Dinner	7.4 (0.2)	6.4 (0.2)	6.0 (0.1)	<0.001
	All Snacks	3.6 (0.1)	3.3 (0.1)	2.6 (0.1)	<0.001

Data presented as means (standard errors), controlled for gender, race/ethnicity, marital status, and income as percent of the federal poverty rate.

**Table 3:** Mean percent intakes from each meal and all snacks by age group.

Intake	Meal	Age Categories (Years)			*p*
		45–59	60–70	≥71	
Energy	Breakfast	17.8 (0.3)	20.4 (0.2)	23.4 (0.4)	<0.001
	Lunch	23.4 (0.3)	22.8 (0.4)	23.2 (0.4)	0.506
	Dinner	35.9 (0.4)	35.5 (0.5)	36.0 (0.5)	0.722
	All Snacks	22.9 (0.4)	21.3 (0.4)	17.4 (0.4)	<0.001
Protein	Breakfast	16.7 (0.3)	19.0 (0.3)	21.3 (0.4)	<0.001
	Lunch	26.7 (0.4)	25.9 (0.5)	26.5 (0.5)	0.368
	Dinner	43.4 (0.5)	42.3 (0.5)	41.6 (0.6)	0.033
	All Snacks	13.2 (0.3)	12.8 (0.4)	10.7 (0.3)	<0.001
Carbohydrate	Breakfast	19.8 (0.3)	22.8 (0.3)	27.2 (0.4)	<0.001
	Lunch	22.3 (0.3)	21.3 (0.4)	21.6 (0.4)	0.082
	Dinner	31.7 (0.4)	31.7 (0.5)	32.1 (0.5)	0.762
	All Snacks	26.2 (0.4)	24.1 (0.4)	19.1 (0.4)	<0.001
Added sugars	Breakfast	22.6 (0.5)	24.9 (0.5)	27.0 (0.6)	<0.001
	Lunch	19.2 (0.4)	17.9 (0.4)	18.7 (0.6)	0.084
	Dinner	23.0 (0.5)	24.4 (0.6)	26.9 (0.6)	<0.001
	All Snacks	35.1 (0.6)	32.9 (0.7)	27.4 (0.7)	<0.001
Dietary fiber	Breakfast	17.8 (0.3)	20.9 (0.3)	26.1 (0.5)	<0.001
	Lunch	25.0 (0.4)	22.9 (0.5)	23.0 (0.4)	<0.001
	Dinner	37.8 (0.5)	37.5 (0.6)	35.9 (0.5)	0.021
	All Snacks	19.4 (0.4)	18.6 (0.4)	15.1 (0.4)	<0.001
Total fat	Breakfast	17.3 (0.3)	19.2 (0.3)	20.6 (0.4)	<0.001
	Lunch	25.1 (0.4)	24.8 (0.5)	24.9 (0.5)	0.886
	Dinner	38.5 (0.5)	37.0 (0.5)	37.7 (0.6)	0.135
	All Snacks	19.2 (0.4)	19.1 (0.5)	16.8 (0.5)	<0.001
Saturated fat	Breakfast	18.4 (0.3)	20.3 (0.3)	21.9 (0.4)	<0.001
	Lunch	23.9 (0.4)	23.8 (0.5)	23.9 (0.5)	0.988
	Dinner	37.7 (0.5)	36.2 (0.6)	36.5 (0.6)	0.128
	All Snacks	19.9 (0.4)	19.6 (0.5)	17.6 (0.5)	0.001
Monounsaturated fat	Breakfast	17.2 (0.3)	19.2 (0.3)	20.3 (0.4)	<0.001
	Lunch	25.3 (0.4)	24.6 (0.5)	24.9 (0.5)	0.574
	Dinner	38.4 (0.5)	37.1 (0.5)	38.0 (0.6)	0.165
	All Snacks	19.1 (0.4)	19.1 (0.5)	16.8 (0.5)	<0.001
Polyunsaturated fat	Breakfast	16.6 (0.4)	18.5 (0.4)	20.1 (0.5)	<0.001
	Lunch	26.6 (0.5)	26.4 (0.6)	26.1 (0.6)	0.803
	Dinner	38.7 (0.5)	37.2 (0.6)	38.4 (0.6)	0.198
	All Snacks	18.1 (0.4)	17.9 (0.5)	15.5 (0.5)	<0.001

Data presented as means (standard errors), controlled for gender, race/ethnicity, marital status, and income as percent of the federal poverty rate.

**Table 4:** Mean macronutrient density of intakes by meals and snack.

Energy-Adjusted (per 1000 kcals)	Meal	Age Categories (Years)			*p*
		45–59	60–70	≥71	
Protein (g)	Breakfast	37.1 (0.5)	38.1 (0.6)	35.5 (0.4)	0.004
	Lunch	47.1 (0.5)	47.5 (0.6)	46.9 (0.7)	0.767
	Dinner	51.1 (0.5)	50.8 (0.6)	48.5 (0.5)	0.001
	All Snacks	22 (0.4)	22.4 (0.5)	22.8 (0.4)	0.336
Carbohydrate (g)	Breakfast	141 (1.0)	142 (1.0)	152 (1.0)	<0.001
	Lunch	117 (1.0)	116 (1.2)	120 (1.3)	0.084
	Dinner	106 (1.0)	108 (1.0)	113 (1.0)	<0.001
	All Snacks	149 (1.0)	149 (2)	149 (1.0)	0.914
Added sugars (g)	Breakfast	39.1 (1.0)	31.7 (0.9)	27.6 (0.7)	<0.001
	Lunch	26.2 (0.7)	21.7 (0.8)	21.7 (1.0)	<0.001
	Dinner	19.8 (0.5)	18.9 (0.5)	20.0 (0.6)	0.211
	All Snacks	56.9 (1.1)	49.7 (1.1)	47.1 (1.1)	<0.001
Dietary fiber (g)	Breakfast	8.8 (0.2)	9.9 (0.3)	11.5 (0.2)	<0.001
	Lunch	9.5 (0.22)	10.1 (0.2)	10.3 (0.2)	0.022
	Dinner	9.1 (0.1)	10.1 (0.2)	10.1 (0.2)	<0.001
	All Snacks	7.9 (0.2)	8.9 (0.2)	9.2 (0.2)	<0.001
Total fat (g)	Breakfast	33.3 (0.4)	32.7 (0.4)	30.8 (0.4)	<0.001
	Lunch	38.7 (0.44)	39.4 (0.55)	38.0 (0.4)	0.095
	Dinner	39.5 (0.3)	38.5 (0.4)	38.3 (0.3)	0.006
	All Snacks	29.9 (0.4)	31.9 (0.5)	32.8 (0.5)	<0.001
Saturated fat (g)	Breakfast	11.6 (0.2)	11.2 (0.2)	10.7 (0.2)	0.002
	Lunch	11.7 (0.1)	11.7 (0.2)	11.6 (0.2)	0.893
	Dinner	12.4 (0.1)	12 (0.2)	11.8 (0.1)	0.008
	All Snacks	10.1 (0.1)	10.7 (0.2)	11.3 (0.2)	<0.001
Monounsaturated fat (g)	Breakfast	12 (0.2)	11.8 (0.2)	10.9 (0.2)	<0.001
	Lunch	13.9 (0.22)	13.9 (0.22)	13.5 (0.22)	0.110
	Dinner	14 (0.1)	13.8 (0.1)	13.7 (0.1)	0.060
	All Snacks	10.8 (0.2)	11.7 (0.3)	12 (0.2)	<0.001
Polyunsaturated fat (g)	Breakfast	6.8 (0.1)	6.9 (0.2)	6.5 (0.1)	0.145
	Lunch	9.5 (0.1)	10.2 (0.2)	9.5 (0.22)	0.026
	Dinner	9.3 (0.1)	9.1 (0.2)	9.2 (0.1)	0.658
	All Snacks	6.4 (0.1)	6.9 (0.2)	6.8 (0.2)	0.042

Data presented as means (standard errors) based on intakes adjusted per 1000 kilocalories of intakes by meal or snacking occasions, controlled for gender, race/ethnicity, marital status, and income as percent of the federal poverty rate.
